# Toll-like receptor 4 promotes the inflammatory response in septic acute kidney injury by promoting p38 mitogen-activated protein kinase phosphorylation

**DOI:** 10.1007/s10863-023-09972-9

**Published:** 2023-08-22

**Authors:** Linlin Yue, Xin Liu, Chaoyu Wu, Jiying Lai, Jie Wang, Huifeng Zhong, Feng Chen

**Affiliations:** 1 Department of Intensive care unit, The First Affiliated Hospital of Gannan Medical University, 128 Jinling Avenue, Zhanggong District, Ganzhou, Jiangxi Province 341000 China; 2 Department of Pediatric Surgery, The First Affiliated Hospital of Gannan Medical University, 128 Jinling Avenue, Zhanggong District, Ganzhou, Jiangxi Province 341000 China; 3 Jiangxi Provincial Clinical Research Center for Vascular Anomalies, The First Affiliated Hospital of GanNan Medical University, Ganzhou, Jiangxi Province 341000 China; 4 Key Laboratory of Prevention and Treatment of Cardiovascular and Cerebrovascular Diseases, Ministry of Education, Gannan Medical University, Ganzhou, Jiangxi Province 341000 China

**Keywords:** Sepsis, Acute kidney injury, Toll-like receptor 4, p38 MAPK, Phosphorylation, Pyroptosis, Inflammatory factor, Inflammatory response

## Abstract

Septic acute kidney injury (AKI) contributes to the mortality and morbidity of sepsis patients. Toll-like Receptor 4 (TLR4) has prominent roles in septic AKI. This study investigated the functions of TLR4 in septic AKI. A septic AKI mouse model was established by cecal ligation and puncture surgery. Mouse kidney function and kidney tissue lesion were examined using corresponding kits and H&E staining. The in vitro cell model of septic AKI was established by lipopolysaccharide induction. Cell viability, inflammatory factor (TNF-α, IL-6, IL-4, IL-1β, IL-18) levels, pyroptotic cell number changes, lactate dehydrogenase (LDH) activity, myeloperoxidase (MOP) concentration, and levels of pyroptosis-associated protein and MyD88, TRIF and p38 MAPK phosphorylation were determined by MTT, ELISA, FAM-FLICA Caspase-1 Detection kit, other corresponding kits, and Western blot. TLR4 was highly expressed in septic AKI mouse kidney tissues and human septic AKI cells. TLR4 knockdown alleviated kidney injury, increased cell viability, and reduced LDH activity and MPO concentration. TLR4 knockdown reduced cell pyroptosis by repressing p38 MAPK phosphorylation through MyD88/TRIF, suppressed pro-inflammatory factor (TNF-α, IL-6, IL-4, IL-1β, IL-18) levels, promoted anti-inflammatory factor (IL-4) level, and reduced inflammatory response, thus playing a protective role in septic AKI. Briefly, TLR4 promoted the inflammatory response in septic AKI by promoting p38 MAPK phosphorylation through MyD88/TRIF.

**Supplementary Information:**

The online version contains supplementary material available at 10.1007/s10863-023-09972-9.

## Introduction

Sepsis is identified as “a life-threatening dysfunction of organ resulting from a dysfunctional response to infection in the hosts” (Huang et al. 2019). Sepsis is still a main cause of mortality and morbidity all over the world, with elevated burden in the middle- and low-resource settings (Salomao et al. 2019). The septic patients are at the risks of secondary injuries, and the aggressive controls of the antibiotic therapy, source, and resuscitation are mainstays of the management (Nunnally 2016). Although sepsis treatment has rapidly developed in the past few years, the incidence and mortality of sepsis in the clinical treatment is still climbing, and due to its diverse manifestations, clinicians still face severe challenges in the diagnosis, treatment, and management of the sepsis patients (Huang et al. 2019). One of the most prevalent organs influenced by sepsis is the kidney, and acute kidney injury (AKI) leads to the mortality and morbidity of sepsis patients (Poston and Koyner 2019). Septic AKI is a prevalent and life-threatening complication in critically ill and hospitalized patients characterized by the rapid deterioration of kidney function related to sepsis, with its pathophysiology incompletely understood, and most treatments remain nonspecific and reactive (Gomez and Kellum 2016). The possible pathogenic mechanisms to explain septic AKI include a dysregulated inflammatory response, microcirculatory dysfunction, and cellular metabolic reprogramming (Manrique-Caballero et al. 2021). Therefore, it is of vital importance to study the mechanism of septic AKI, so as to provide efficient treatment for it.

Toll-like receptors (TLRs) are critical for sensing invading microorganisms like virus, bacteria, and fungi, which as a first line of defense, initiate an innate immune response that includes macrophage, monocyte and polymorphnuclear leukocyte activation, and also mediate pro-inflammatory cytokine release and interferons with the ultimate goal of identifying and destroying pathogen (Anderberg et al. 2017). Particular attention has been paid to TLR4, the receptor for the Gram-negative bacteria outer membrane lipopolysaccharide (LPS) or endotoxin, and the therapeutic targeting of TLR4 in sepsis, because of its essential role, looks promising (Wittebole et al. 2010). Pyroptosis is a programmed cell death with caspase-1 dependence involved in inflammation that is initiated by cytosolic LPS or inflammasomes in innate immunity, which has a crucial property in sepsis (Liu and Sun 2019). Mitogen-activated protein kinase (MAPK) is the crucial component of the signaling pathways in cells that presents receptor signals in the nucleus on the cell surface to the DNA, and the repression of the p38 MAPK pathway suppresses macrophage pyroptosis and decreases inflammatory factor release in acute lung injury (Zhou et al. 2019). However, whether TLR4 promoted the inflammatory response in septic AKI by promoting p38 MAPK phosphorylation remained largely unknown. We aimed to study TLR4 mechanism in septic AKI to provide some reference value for its treatment.

## Materials and methods

### Ethics statement

All procedures were authorized by the academic ethics committee of The First Affiliated Hospital of Gannan Medical University. All procedures were strictly implemented according to the guide of the National Institutes of health. All the laboratory procedures were used to reduce the pain of the mice.

### Experimental animals

Male C57BL/6 mice (6–8 weeks old, weighing 20–25 g) were purchased from Vital River Laboratories [SYXK (Beijing) 2016-0011, Beijing, China]. The mice were raised under standard temperature and humidity, in a 12-h light/dark cycle, with adequate food and water.

### Animal model establishment

A total of 24 male C57BL/6 mice (6–8 weeks old, weighing 20–25 g) were fasted for 12 h, and anesthetized by intraperitoneal injection of 2.5% pentobarbital sodium (2 mL/kg) before surgery. The mice were assigned into 4 groups by random digit table, with 6 in each group: sham group, AKI group, AKI + sh-NC group and AKI + sh-TLR4 group. The treatment of each group was as follows: sham group: the distal end of the cecum and mesentery was separated by surgical laparotomy, and abdominal closure was performed; AKI group: mice were treated by CLP surgery to induce high-grade sepsis. In short, the abdomen was routinely disinfected and a 2-cm incision was made in the middle of abdomen to expose the cecum. Next, the distal end of the cecum and mesentery were separated to avoid damaging the mesenteric vessels. Subsequently, sterile suture 4 was used for ligation at 3/4 of the distal end of the cecum, and a sterile No. 7 pipette was used to puncture the cecum in a single pass midway between the ligation and the cecal tip, and finally the abdomen was sutured layer by layer; AKI + sh-NC/TLR4 group: after 4 h of CLP surgery, mice were injected with 1.5 × 10^9^ TU sh-NC/TLR4 lentiviral vector through tail vein. After 48-h injection, mice were euthanized by the excessive pentobarbital sodium (800 mg/kg; P3761, Sigma-Aldrich, Louis, MO, USA), and kidney tissues and blood samples were collected for further analysis. sh-NC/TLR4 was purchased from GenePharma (Shanghai, China). The sequence of TLR4 shRNA was 5’-aaCATCTGGATTTCCAGCAATTTCAAGAGAAATGCTGGAAATCCAGATGtt-3’.

### Tissue biochemical analysis and pathological detection

Blood samples were collected and centrifuged to obtain mouse serum samples, and serum creatinine (Scr) and blood urea nitrogen (BUN) levels were analyzed using an Olympus AU400 automatic chemical analyzer (Olympus). Parameters were set in accordance with instrument instructions. The samples and reagent were placed on the instrument to read data.

Mouse kidney tissues were collected, fixed with 4% formaldehyde for 6 h, embedded in paraffin, cut into sections, dewaxed with xylene, and soaked in 100%, 95%, 80% and 75% ethanol. The sections were washed with distilled water, stained for 10 min with hematoxylin staining solution, washed with distilled water, differentiated with differentiation solution for 30 s, soaked in distilled water for 15 min, stained with eosin staining solution for 2 min, washed with distilled water, soaked in 95%, 95%, 100% and 100% ethanol for 1 min, immersed in xylene carbonate (3:1) for 1 min, in xylene (I) for 1 min, and in xylene (II) for 1 min, and sealed with neutral resin. The samples were observed and randomly imaged using a microscope (Olympus, Tokyo, Japan). Referring to the scoring system applied in previous studies (Kurus et al. 2009; Tanuseputero et al. 2020), renal tubulointerstitial injury was scored based on the scope of renal tubular injury (including dilation, atrophy, epithelial cell vacuolar degeneration, and necrotic renal tubules), with 0 indicating no tubular injury, 1 indicating < 10% of tubules injured, 2 indicating 10-25% of tubules injured, 3 indicating 26-50% of tubules injured, 4 indicating 51-75% of tubules injured, and 5 indicating > 75% of tubes injured.

### Cell culture and treatment

Human tubular epithelial cell line (HK-2) was maintained in the laboratory and incubated at 37 °C containing 5% CO_2_ in the Roswell Park Memorial Institute (RPMI)-1640 medium (GE, Madison, WI, USA) containing fetal bovine serum (15%, GE), streptomycin (0.1 mg/mL, GE) and penicillin (100 units/mL, GE). To induce a septic AKI cell model, HK-2 cells were treated with lipopolysaccharide (LPS, 1 mg/L, Sigma-Aldrich) for 24 h after the cell concentration reached 80%. After that, the obtained shRNA lentiviral vector carrying TLR4 (GenePharma, Shanghai, China) and its negative control sh-NC, p38 MAPK activator U-46,619 (Calbiochem, San Diego, CA, USA) and its control dimethyl sulfoxide (DMSO) were introduced into HK-2 cells (cultured to a confluence of more than 80%) using Lipofectamine 3000 (Thermo, San Jose, CA, USA). After 48-h transfection, subsequent experiments were performed.

HK-2 cells were allocated into 6 groups. control group, LPS group, LPS + sh-NC group, LPS + sh-TLR4 group, LPS + sh-TLR4 + DMSO group, LPS + sh-TLR4 + MA (MAPK activator, 10 µM) group. All cells except cells in the control group were treated with LPS (Sigma-Aldrich) at a concentration of 1 µg/mL for 24 h, and cells in the control group were treated with an equivalent volume of phosphate-buffered saline for 24 h.

### 3-(4,5-dimethylthiazol-2-yl)-2,5-diphenyl-2 H-tetrazolium bromide (MTT) assay

Cells were seeded at 5 × 10^3^ cells/well in the 96 well plates. After 24-h incubation at 37 °C, cells were supplemented with 20 µL (5 g/L) MTT in each well and incubated for another 4 h. After that, the medium was removed and 150 µL DMSO was supplemented to each well. Finally, the optical density (OD) value at 570 nm was determined using a microplate reader (Bio-Rad Laboratories, Shanghai, China) for cell viability detection.

### Inflammatory factor determination by enzyme-linked immunosorbent assay (ELISA)

Whole blood samples were collected using anticoagulant free tubes and centrifuged for 10 min at 1000 g at 4 °C, and the supernatant was stored at -80 °C for further analysis. The cell culture supernatant was collected and centrifuged at 1000 g for 5 min at 4 °C. Levels of tumor necrosis factor (TNF)-α, interleukin (IL)-6, IL-1 β, IL-18 and IL-4 were measured using a commercial ELISA kit (HS Quantikine; R&D Systems, Minneapolis, MN, USA) following manufacturer’s guidelines.

### Reverse transcription quantitative polymerase chain reaction (RT-qPCR)

Total RNA was extracted from HK-2 cells and kidney tissues using the Trizol reagent (Invitrogen, Carlsbad, CA, USA). The obtained RNA was reverse-transcribed using the PrimeScript RT kit (Takara, Tokyo, Japan) to synthesize cDNA. RT-qPCR was performed using the SYBR Green I Master Mix kit (Invitrogen, Thermo Fisher Scientific) on the 7300 real-time PCR system (Applied Biosystems; Thermo Fisher Scientific) for TLR4 level measurement. All procedures were performed following manufacturer’s instructions. With GAPDH as the internal reference, the relative expression was calculated using the 2^-ΔΔCt^ method (Livak and Schmittgen 2001). Primer sequences are shown in Table 1.

**Table 1:** Primer sequences

Gene	sequences
Mouse, TLR4	F: 5’-CTGGGTGAGAAAGCTGGTAA-3’
	R: 5’-AGCCTTCCTGGATGATGTTGG-3’
Mouse, GAPDH	F: 5’-GACATCAAGAAGGTGGTGAAGC-3’
	R: 5’-GAAGGTGGGAAGAGTGGGAGTT-3’
Human, TLR4	F: 5’-TGGAAGTTGAACGAATGGAATGTG-3’
	R: 5’-ACCAGAACTGCTACAACAGATACT-3’
Human, GAPDH	F: 5’-GGCACAGTCAAGGCTGAGAATG-3’
	R: 5’-ATGGTGGTGAAGACGCCAGTA-3’

### Western blot

Cells or homogenated tissues were obtained and lysed in the lysis buffer containing 250 mM sucrose (Duchefa Biochemie, 57-50-1), 50 mM NaCl, 20 mM Tris-HCl, pH 7.4, 1 mM ethylenediamine tetraacetic acid, 1% Triton X-100, 1 × protease, and incubated with a mixture of phosphate inhibitors (Thermo Fisher Scientific, 78,440), 1 mM dithiothreitol, and 1 mM phenylmethylsulfonyl fluoride on ice for 15 min. After incubation, lysates were centrifuged (16,100 × g, 10 min) and the supernatant was collected. Protein concentration was measured using the bicinchoninic acid protein assay reagent (Thermo Fisher Scientific, 23,225). Samples were separated by sodium dodecyl sulfate gel electrophoresis, electrotransferred to the polyvinylidene fluoride membranes in a semi-dry electrophoretic transfer cell (Bio-Rad), and blocked for 1 h with 5% skim milk powder dissolved in Tris buffered saline (20 mM Tris base, 0.5 M NaCl, pH 7.5) containing 0.1% Tween-20 (TBST) at room temperature. The membranes were then incubated with the primary antibodies NLRP3 (1:1000, ab263899, Abcam, Cambridge, MA, USA), pro-Caspase-1 (p45) (1:1000, 22915-1-AP, Proteintech, Rosemont, IL, USA), Caspase1 (p10) (1:1000, 221,312, AdipoGen, San Diego, CA, USA), Caspase-1 (p20) (1:1000, 144,921, AdipoGen), GSDMD-N (1:1000, ab219800, Abcam), MyD88 (1:1000, ab219413, Abcam), TRIF (1:1000, ab302562, Abcam), p-p38 (1:1000, #4511, CST, Danvers, MA, USA), p38 (1:1000, #8690, CST), β-Tubulin (1:1000, #2146, CST) in a shaking incubator at 4 °C overnight. After that, samples were washed 3 times with TBST (each time for 10 min), and incubated for 1 h with peroxidase conjugated secondary antibody diluted in the blocking solution at room temperature. After washing, the results were observed after developing and fixing. Western blot images were analyzed using ImageJ2x V2.1.4.7 (Rawak Software, Germany) software.

### FAM-FLICA Caspase-1 detection

FAM-FLICA Caspase-1 Detection kit (ImmunoChemistry, Bloomington, MN, USA) was adopted in FAM-YVAD-FMK and propidium iodide (PI) double staining for pyroptosis cell number evaluation. FAM-YVAD-FMK (green), PI (red), Hoechst 33,342 (blue).

### Lactate dehydrogenase (LDH) assay

The activity of LDH in the culture medium was measured using The LDH assay kit (K313; biovision, Tucson, AZ, USA) to evaluate cell damage. The 10 µL medium was collected and analyzed using LDH detection kit.

### Myeloperoxidase (MPO) assay

Kidney tissue samples were mixed with buffer solution (1:19) using a tissue homogenizer (Jingxin, Shanghai, China) to prepare 5% homogenate. MPO concentration was measured using a commercial kit (Jiancheng Bioengineering Institute, Nanjing, Jiangsu, China) for neutrophil accumulation evaluation following manufacturer’s instructions.

### Statistical analysis

SPSS 22.0 (IBM Corp. Armonk, NY, USA) and GraphPad Prism 8.0 (GraphPad Software Inc., San Diego, CA, USA) were applied for statistical analysis and mapping. Measurement data were expressed by mean ± standard deviation. Variance normality and homogeneity were tested. Data were in normal distribution with uniform variance. Data between groups were compared by independent *t* test. Data among groups were compared by one-way analysis of variance (ANOVA), followed by Tukey’s multiple comparisons test. The *P* value was obtained by a bilateral test. *P* < 0.05 indicated statistical significance.

## Results

### Knockdown of TLR4 had protective effects on septic AKI mice

**Fig. 1:**
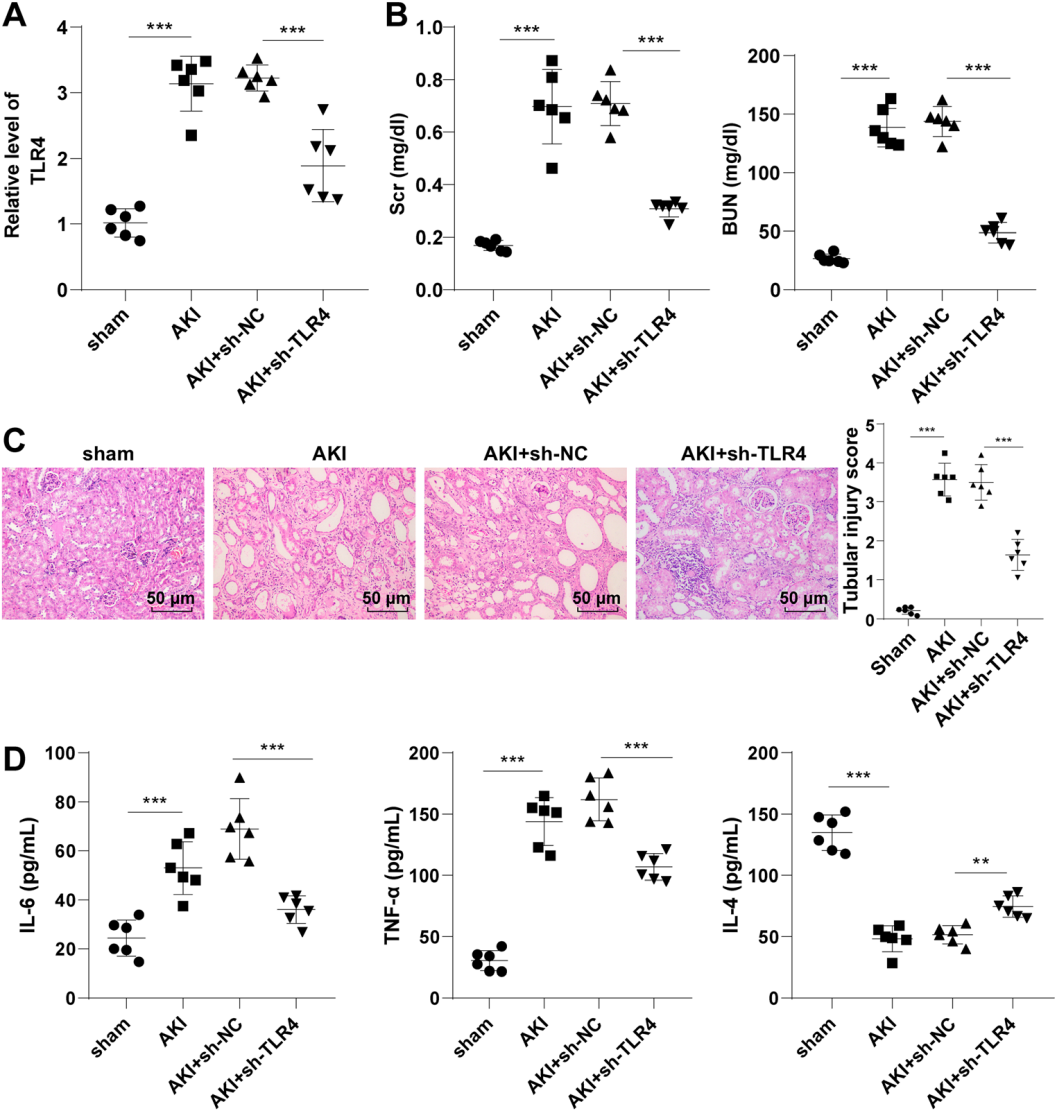
TLR4 knockdown had protective effects on septic AKI mice. With sh-NC as the control, sh-TLR4 was transfected into the AKI mouse model. **A:** TLR4 expression in mice was measured by RT-qPCR; **B:** The concentrations of serum and urea nitrogen in mice were determined using an automatic biochemical instrument; **C:** The morphology of mouse kidney tissues was observed by H&E staining, and renal tubulointerstitial injury was scored based on the extent of involvement of the injuried renal tubules; **D:** The levels of serum inflammatory factors were determined by ELISA. N = 6. Data were expressed by mean ± standard deviation. Data among groups were compared by one-way ANOVA, followed by Tukey’s multiple comparisons test. ** *P* < 0.01, *** *P* < 0.001

A septic AKI mouse model was established by CLP surgery to explore the function of TLR4. RT-qPCR manifested that TLR4 was highly expressed in the kidney of AKI mice. After sh-TLR4 was introduced into AKI mice, TLR4 expression was repressed (all *P* < 0.001, Fig. 1A), indicating that the transfection was successful. Scr and BUN expression patterns in the AKI group were elevated, while the AKI + sh-TLR4 group showed the opposite trend (all *P* < 0.001, Fig. 1B). In H&E staining, compared with the sham group, renal tubular epithelial cells in the AKI group shed, and brush border and renal epithelial cells were decreased, while knockdown of TLR4 improved the pathological damage of renal tissues (Fig. 1C). In addition, the levels of serum IL-6 and TNF-α in the AKI group were raised, and the level of IL-4 was suppressed (all *P* < 0.001, Fig. 1D), while TLR4 silencing reduced the expression patterns of the inflammatory factors in AKI mouse model. These phenomena suggested that TLR4 silencing had a protective effects on septic AKI mice.

### TLR4 silencing exerted protective effects on septic AKI mice by inhibiting cell pyroptosis

**Fig. 2:**
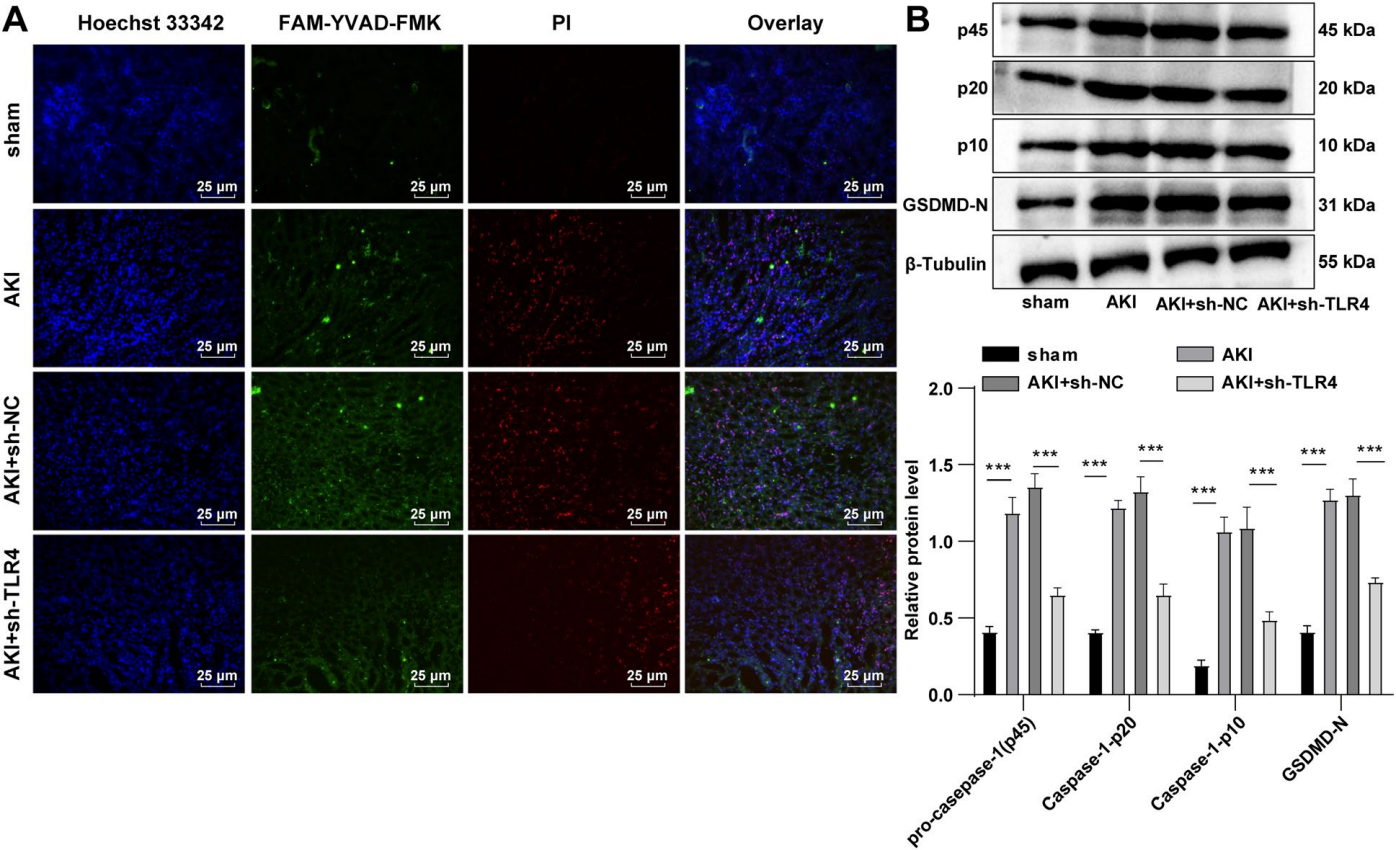
TLR4 silencing exerted protective effects on sepsis AKI mice by inhibiting cell pyroptosis. sh-TLR4 was transfected into AKI mouse model, and sh-NC was used as a control. **A:** The number of pyroptosis cells in tissues was counted using the FAM-FLICA Caspase-1 Detection kit; **B:** The expression levels of pyroptosis-related proteins Caspase-1 (p45, p20 and p10) and GSDMD-N were determined by Western blot. N = 6. Data were expressed by mean ± standard deviation. Data among groups were compared by one-way ANOVA, followed by Tukey’s multiple comparisons test. *** *P* < 0.001

Pyroptosis aggravates the pathological damage of sepsis-induced AKI, and inhibiting pyroptosis can alleviate the kidney injury (Lin et al. 2020). Therefore, we speculated that the protective effects of TLR4 silencing on sepsis-induced AKI mice might be achieved by regulating cell pyroptosis. Pyroptotic cell number in the AKI group was increased, while the number of pyroptotic cells in the AKI + sh-TLR4 group was decreased (Fig. 2A). Western blot demonstrated that the expression patterns of Caspase-1 (p45, p20 and p10) and GSDMD-N was facilitated in the AKI group and repressed in the AKI + sh-TLR4 group (all *P* < 0.01, Fig. 2B). These results suggested that the protective effects of TLR4 silencing on septic AKI might be achieved by regulating cell pyroptosis.

### TLR4 silencing inhibited cell pyroptosis

**Fig. 3:**
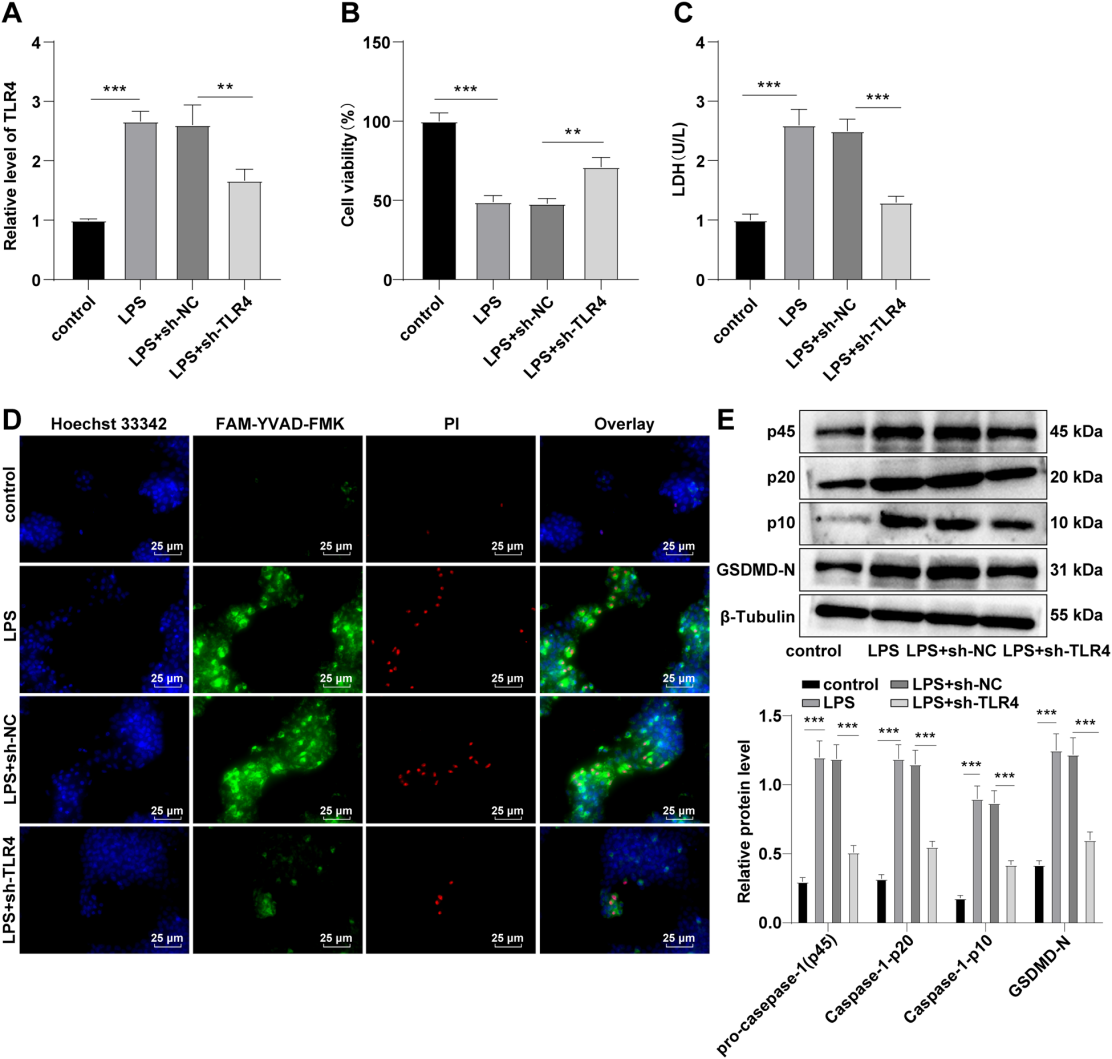
TLR4 knockdown inhibited cell pyroptosis. LPS-induced HK-2 cells were used to establish an AKI cell model, and sh-TLR4 was transfected into the cells, with sh-NC as the control. **A:** The expression of TLR4 was determined by RT-qPCR; **B:** Cell viability was detected by MTT ; **C:** The LDH activity was detected using the kit; **D:** The number of pyroptosis cells was counted by FAM-FLICA Caspase-1 detection kit; **E:** The expression patterns of pyroptosis-related proteins Caspase-1 (p45, p20 and p10) and GSDMD-N were measured by Western blot. Cell experiment was repeated 3 times. Data were expressed by mean ± standard deviation. Data among groups were compared by one-way ANOVA, followed by Tukey’s multiple comparisons test. ** *P* < 0.01, *** *P* < 0.001

In the previous experiments, we demonstrated in vivo that TLR4 silencing exerted protective effects on septic AKI mice by inhibiting cell pyroptosis. The in vitro validation experiments were further carried out. HK-2 cells were induced with LPS at a concentration of 1 µg/mL for 24 h, and septic AKI cell models were established. RT-qPCR revealed that compared with the control group, TLR4 expression in the LPS group was upregulated (*P* < 0.001, Fig. 3A). RT-qPCR demonstrated that after sh-TLR4 transfection, TLR4 expression was repressed (*P* < 0.01, Fig. 3A), indicating that the transfection was successful. The cell viability and LDH activity were assessed. Compared with the control group, cell viability was blocked and LDH activity was augmented in the LPS group, while the inhibition of TLR4 increased cell viability and decreased cell LDH activity (all *P* < 0.01, Fig. 3B-C). The number of LPS-treated cell pyroptosis was augmented, while TLR4 silencing inhibited the increase in the number of cell pyroptosis (Fig. 3D). Western blot elucidated that the Caspase-1 (p45, p20 and p10) and GSDMD-N expression levels were stimulated in the LPS group and suppressed in the LPS + sh-TLR4 group (all *P* < 0.01, Fig. 3E). The results suggested that TLR4 silencing inhibited cell pyroptosis and alleviated cell damage.

### TLR4 knockdown inactivated NLRP3 inflammatory receptors and reduced inflammatory factor expression levels

**Fig. 4:**
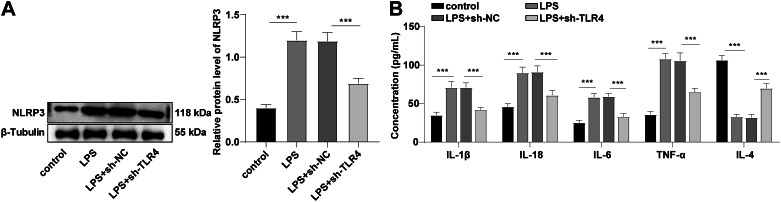
TLR4 knockdown inhibited the activation of NLRP3 inflammatory receptors and reduced the expression levels of inflammatory factors. LPS-induced HK-2 cells were used to establish an AKI cell model, and sh-TLR4 was transfected into the cells, with sh-NC as the control. **A:** The expression of NLRP3 was measured by Western blot; **B:** IL-1β, IL-18, IL-6, TNF-α and IL-4 levels were determined by ELISA. Cell experiment was repeated 3 times. Data were expressed by mean ± standard deviation. Data among groups were compared by one-way ANOVA, followed by Tukey’s multiple comparisons test. *** *P* < 0.001

Pyroptosis is a inflammation-dependent programmed cell death involved in inflammasome activation (Zeng et al. 2020). NLRP3 inflammasome is a well-known inflammasome mainly found in monocytes and macrophages and can mediate kidney injury (Hutton et al. 2016). Western blot elicited that NLRP3 expression in the LPS group was facilitated, while knockdown of TLR4 reduced NLRP3 expression in the LPS group (all *P* < 0.001, Fig. 4A). ELISA exhibited that IL-1β, IL-18, IL-6 and TNF-α expression patterns in the LPS group was augmented, and IL-4 expression was diminished, while knockdown of TLR4 blocked the LPS-triggered changes of inflammatory factors (all *P* < 0.001, Fig. 4B). Briefly, TLR4 silencing in activated NLRP3 inflammatory receptors and reduced the expression levels of inflammatory factors.

### TLR4 activated p38 MAPK phosphorylation, mediated cell pyroptosis and promoted inflammatory response via the MyD88/TRIF-dependent transduction pathway

**Fig. 5:**
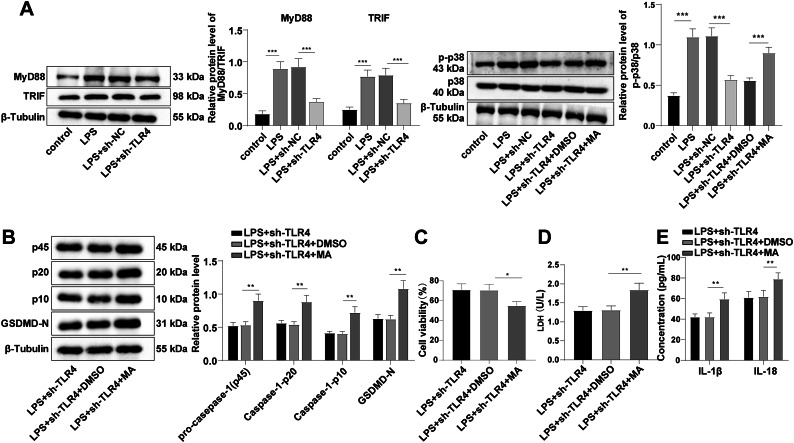
TLR4 activated p38 MAPK phosphorylation, mediated cell pyroptosis and promoted inflammatory response via the MyD88/TRIF pathway. p38 MAPK activator (U-46,619) was added to sh-TLR4 transfected and LPS-stimulated HK-2 cells, with DMSO as the control. **A:** MyD88, TRIF, and p38 MAPK phosphorylation level, NLRP3 protein level **B:** Expression levels of pyroptosis-related proteins Caspase-1 (p45, p20 and p10) and GSDMD-N were determined by Western blot; **C:** Cell viability was detected by MTT; **D:** The LDH activity was detected using the kit; **E:** The expression levels of inflammatory factors were assessed by ELISA. Note: MA, p38 MAPK activator. Cell experiment was repeated 3 times. Data were expressed by mean ± standard deviation. Data among groups were compared by one-way ANOVA, followed by Tukey’s multiple comparisons test. * *P* < 0.05, ** *P* < 0.01, *** *P* < 0.001

The TLR4 signaling is classified into the MyD88-dependent and MyD88 independent (TRIF-dependent) pathways. P38 MAPK plays a critical role in regulating cell growth, proliferation, differentiation and inflammation (Pearson et al. 2001; Sui et al. 2014). In animals with LPS-induced acute lung injury, p38 MAPK expression is up-regulated (Ma et al. 2015). We speculated that TLR4 promoted inflammatory response by regulating cell pyroptosis through MyD88/TRIF/p38 MAPK. Western blot manifested that compared with the control group, MyD88 and TRIF protein levels and the phosphorylation of p38 MAPK in the LPS group were all stimulated, while TLR4 inhibition reversed this phenomenon (all *P* < 0.01, Fig. 5A). The addition of p38 MAPK activator partially annulled the sh-TLR4-mediated decrease of NLRP3, Caspase-1 (p45, p20 and p10) and GSDMD-N (all *P* < 0.01, Fig. 5B). MTT revealed that cell viability was blocked by p38 MAPK activator (all *P* < 0.01, Fig. 5C). In addition, p38 MAPK activator stimulated LDH activity and the levels of inflammatory factors IL-1β and IL-18 (all *P* < 0.01, Fig. 5D-E). These results suggested that TLR4 activated p38 MAPK phosphorylation, mediated pyroptosis and promoted inflammatory response via the MyD88/TRIF pathway.

### Knockdown of TLR4 inhibited p38 MAPK phosphorylation and inflammatory response in septic AKI mice

**Fig. 6:**
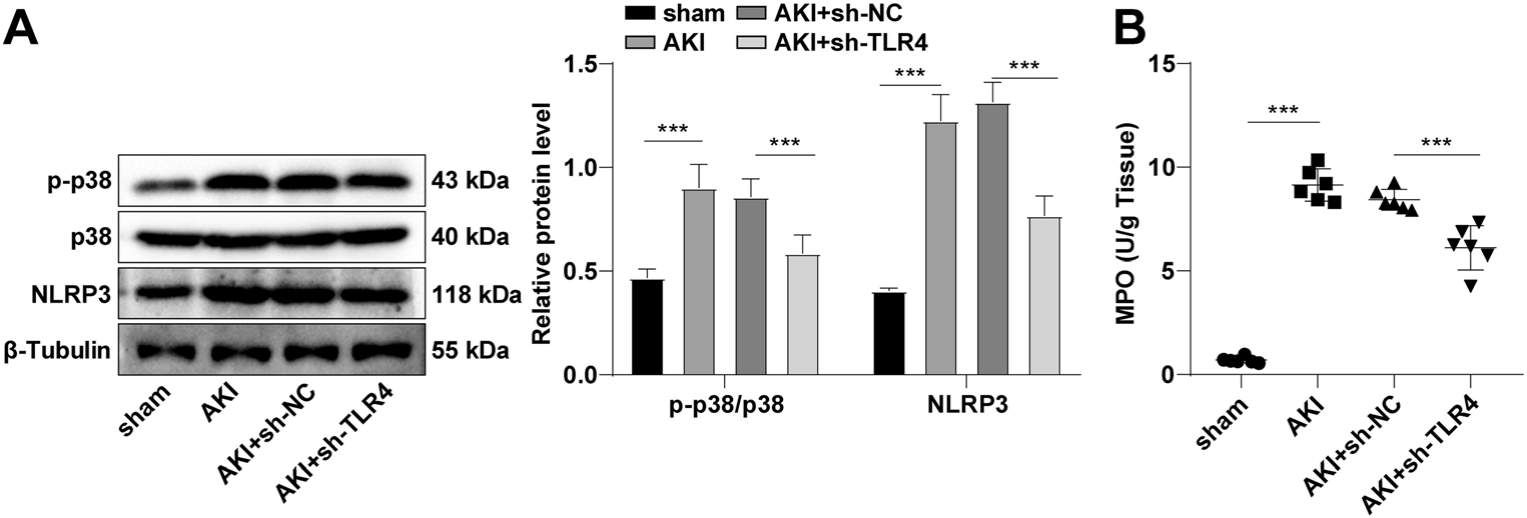
Knockdown of TLR4 inhibited p38 MAPK phosphorylation and inflammatory response in septic AKI mice. sh-TLR4 was transfected into AKI mouse model, with sh-NC as the control. **A:** p38 MAPK phosphorylation and NLRP3 protein expression were determined by Western blot; **B:** MPO concentration was determined using the kit. N = 6. Data were expressed by mean ± standard deviation. Data among groups were compared by one-way ANOVA, followed by Tukey’s multiple comparisons test. *** *P* < 0.001

Inflammatory cell infiltration and changes in proinflammatory cytokines have a crucial property in the pathological process of sepsis (Michie et al. 1988). To verify that knockdown of TLR4 inhibited p38 MAPK phosphorylation and suppressed inflammatory response in septic AKI mice in vivo, p38 MAPK phosphorylation and NLRP3 protein expression in kidney tissues were determined. Western blot demonstrated that p38 MAPK phosphorylation level and NLRP3 expression were decreased after TLR4 knockdown (all *P* < 0.01, Fig. 6A). MPO, as an index of neutrophil infiltration in sepsis, has consistent patterns with inflammatory cytokines. Compared with the sham-operated mice, MPO activity in AKI mice was facilitated, while TLR4 knockdown reduced MPO activity (all *P* < 0.01, Fig. 6B). The results suggested that knockdown of TLR4 inhibited p38 MAPK phosphorylation and suppressed inflammatory response in septic AKI mice.

## Discussion

Sepsis and septic shock are prevalent life-threatening pathological conditions related to high mortality and substantial costs in the healthcare system (Esposito et al. 2017). The most severe form of sepsis leads to multiple organ dysfunction, which can cause a state of chronic critical illness that is characterized by severe catabolism and immune dysfunction (Gotts and Matthay 2016). Septic AKI is a common complication in the critically ill patient, which is related to the unacceptable morbidity and mortality (Peerapornratana et al. 2019). Evidence has shown that TLR4 is involved in the inflammation and kidney injury in LPS-induced septic AKI (Zhong et al. 2020). This study discovered that TLR4 promoted the inflammatory response in septic AKI by promoting p38 MAPK phosphorylation.

Kidney injury is characterized by elevated Scr and BUN levels (Xu et al. 2020). Pro-inflammatory factors TNF-α and IL-6 are augmented and anti-inflammatory factor IL-4 is suppressed in septic AKI rats (Li et al. 2020). Our results revealed that AKI mice manifested with raised Scr and BUN levels, exfoliation of tubular epithelial cells, reduced brush border and renal epithelial cells, promoted serum IL-6 and TNF-α levels and blocked IL-4 level, while the mice transfected with sh-TLR4 showed the opposite trends. It is consistent with the finding that TLR4 inhibitor suppresses Scr and BUN levels, and reduces tubular apoptosis and morphological injury in iopromide-induced AKI (Tan et al. 2017). Quercetin inhibits TNF-α and IL-6 production in LPS-induced AKI mice by inhibiting TLR4 (Guo et al. 2020). In summary, inhibition of TLR4 had effects on protecting septic AKI mice.

Pyroptosis, as a kind of inflammatory-modulated cell death, is involved in a variety of inflammatory diseases, including AKI (Xia et al. 2021). Kidney function loss, cell death and tissue damage induced by cisplatin can be alleviated by decreased expression patterns of GSDMD-N and Caspase-1 (features of pyroptosis) (Jiang et al. 2021). Our results manifested that AKI mice showed increased pyroptotic cell number, and Caspase-1 (45, p20, p10) and GSDMD-N protein levels, while knockdown of TLR4 decreased these levels. Pyroptosis causes cell lysis and inflammatory cytokine release, and cell lysis leads to intracellular content release, including LDH (Kesavardhana and Kanneganti 2017; Xu et al. 2018). Similarly, LPS-induced HK-2 cells demonstrated blocked cell viability and LDH activity, and augmented pyroptotic cell number and Caspase-1 (45, p20, p10) and GSDMD-N protein levels, while knockdown of TLR4 abrogated these trends. Consistently, ibudilast decreases the levels of pyroptosis-related proteins (caspase-1 and GSDMD cleavage) by inhibiting folic acid-induced TLR4 upregulation in AKI (Li et al. 2021). In conclusion, knockdown of TLR4 had effects on protecting septic AKI mice or LPS-induced HK-2 cells by modulating cell pyroptosis.

NLRP3 inflammasome has been implicated in the pathogenesis of various kidney conditions, including AKI, which converts the pro-inflammatory cytokines to their active forms in response to the danger signals that can be either pathogen or host derived, and induces a inflammatory cell death form called pyroptosis (Hutton et al. 2016). The biochemical function of inflammasomes is to activate Caspase-1, which leads to IL-1β and IL-18 maturation and pyroptosis induction (He et al. 2016). Our results manifested that after LPS induction, NLRP3 expression was facilitated, and IL-1β, IL-18, IL-6 and TNF-α expression levels were stimulated, and IL-4 expression was suppressed, while these trends were opposite after TLR4 silencing. It is consistent with that dexmedetomidine alleviates LPS-induced AKI by inhibiting the activation of the NLRP3 inflammasomes and IL-18 and IL-1β levels via inhibiting the TLR4/NOX4/NF-κB pathway (Yao et al. 2019). To conclude, TLR4 silencing inactivated the NLRP3 inflammatory receptors and changed the trends of inflammatory factors. The activation of the MAPK pathway is responsible for the excessive generation of the proinflammatory cytokines (Ma et al. 2015). MPO is a lysosomal enzyme that is involved in the oxidative stress-mediated kidney injury (Perianayagam et al. 2012). The TLR signaling pathway includes the MyD88-dependent and MyD88 independent transduction pathways (TRIF-dependent pathway), and TLR4 can be transduced through both of the MyD88-dependent and TRIF-dependent pathways. The MyD88-dependent pathway is a classic pathway of signal transduction that primarily stimulates downstream inflammatory effects by mediating the production of NF-κB and pro-inflammatory cytokines. The TRIF-dependent pathway activates interferon factor 3 and induces transcription of IFNβ by forming complexes with TRAM. Our results demonstrated that LPS induction elevated MyD88 and TRIF protein levels and p38 MAPK phosphorylation level, and the level was decreased by TLR4 silencing. Furthermore, we added p38 MAPK activator U-46,619 into the LPS-induced cells transfected with sh-TLR4 and discovered that the inhibition of NLRP3, Caspase-1 (p45, p20, p10), and GSDMD-N expression patterns by sh-TLR4 was partially averted, cell viability was blocked, and LDH activity and IL-1β and IL-18 expression levels were enhanced. In vivo experiments revealed the similar results that after TLR4 was knocked down, the phosphorylation of p38 MAPK and NLRP3 expression in kidney tissues, and MPO activity were decreased. Consistently, the activation of TLR4 in kidney parenchymal cells activates the p38 MAPK pathways, causing facilitated production of the inflammatory cytokines, and subsequent kidney injury (Zhang et al. 2008). In brief, TLR4 activated the phosphorylation of p38 MAPK, mediated pyroptosis and promoted inflammatory response, and TLR4 silencing inhibited the phosphorylation of p38 MAPK, and inhibited inflammatory response in septic AKI mice via the MyD88/TRIF pathway.

In summary, this study supported that TLR4 aggravated the inflammatory response in septic AKI by promoting the p38 MAPK phosphorylation and mediating cell pyroptosis. This mechanism was currently less reported, and our study provided certain reference value for the treatment of septic AKI patients. However, we only simply revealed that TLR4 silencing exerted a protective mechanism on septic AKI patients by regulating p38 MAPK phosphorylation and cell pyroptosis, and didn’t accurately determine the direct role of TLR4 in clinical treatment of septic patients in depth. In the future, it is necessary to carry out further in-depth clinical research to accurately identify the therapeutic effect of TLR4 on septic AKI patients.

### Electronic supplementary material

Below is the link to the electronic supplementary material.

Supplementary Material 1

## Data Availability

The datasets used and analysed during the current study are available from the corresponding author on reasonable request.
