# Supplementary figures and images for: Toll-like receptor 4 promotes the inflammatory response in septic acute kidney injury by promoting p38 mitogen-activated protein kinase phosphorylation
Source: J Bioenerg Biomembr. 2023 Aug 22;55(5):353–63. doi: 10.1007/s10863-023-09972-9 (PMC10556113; doi:10.1007/s10863-023-09972-9)

2B

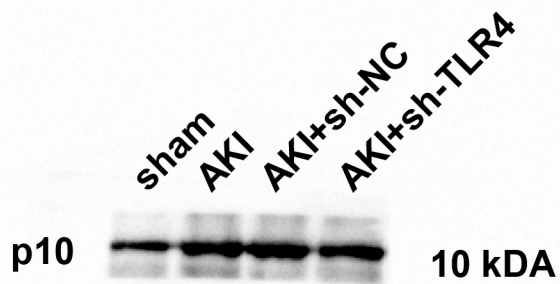

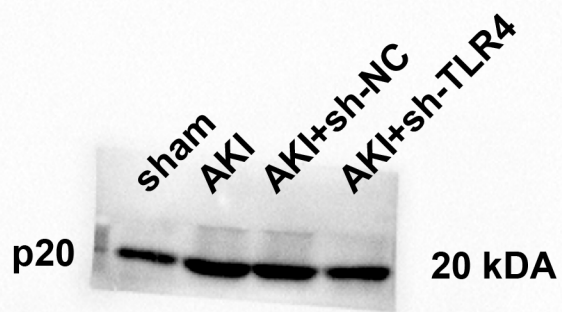

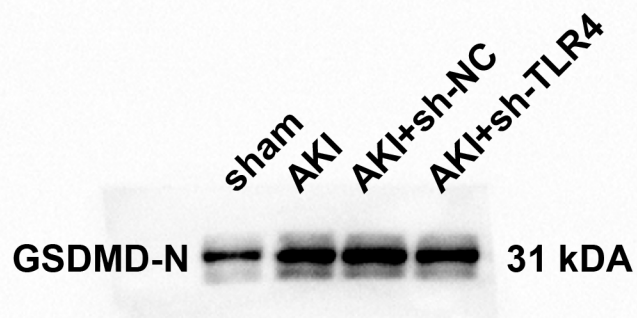

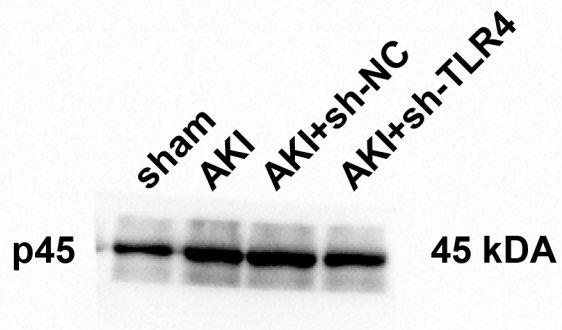

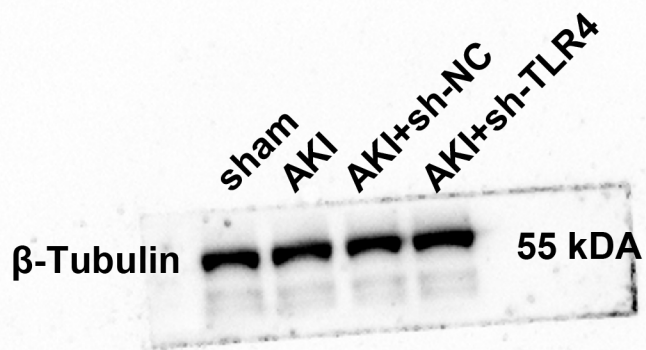

3E

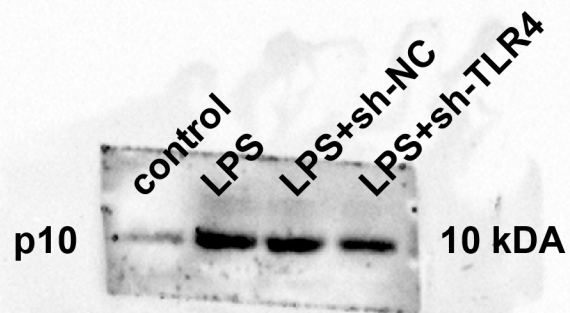

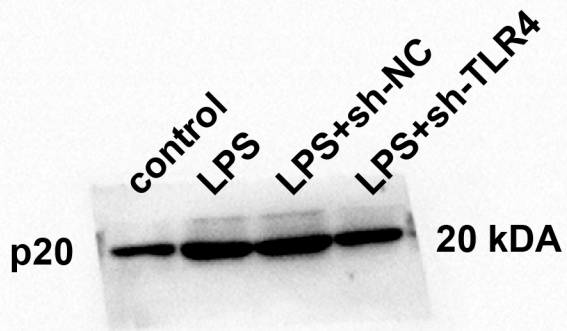

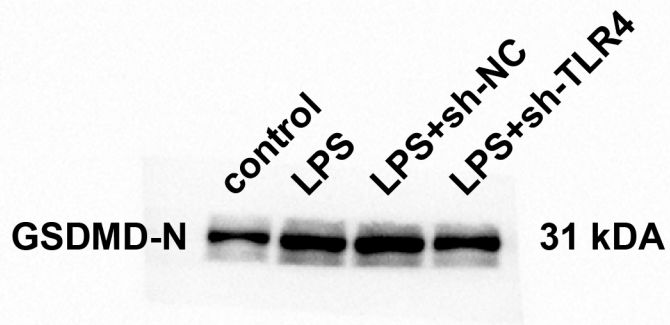

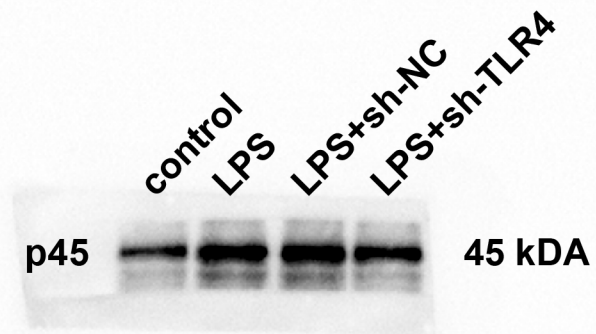

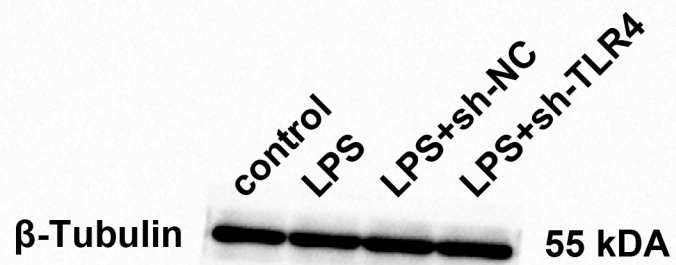

4A

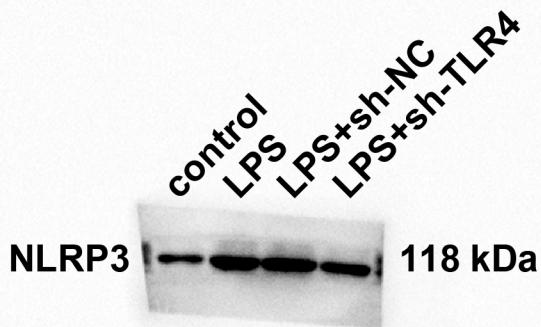

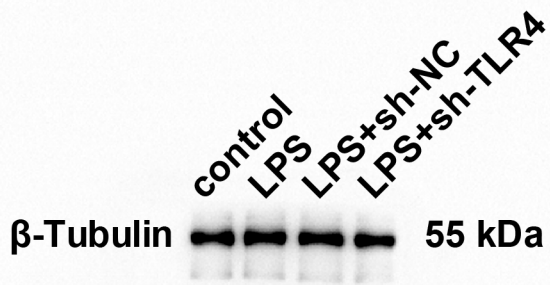

5A

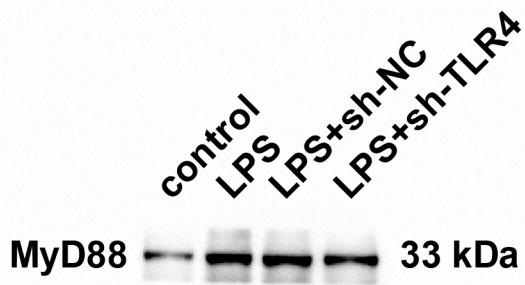

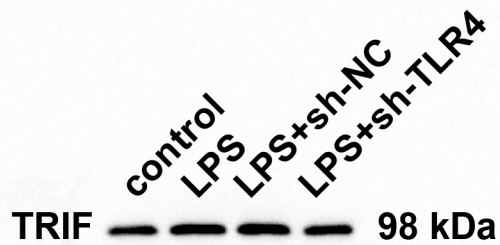

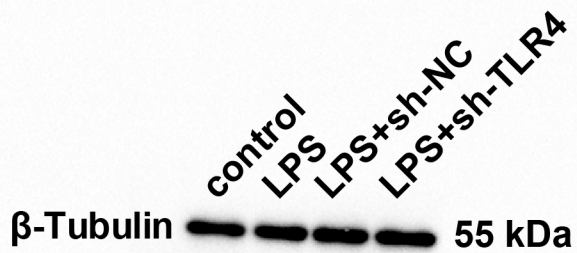

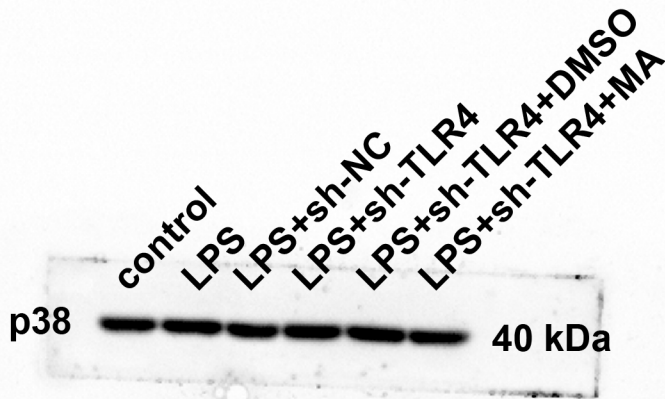

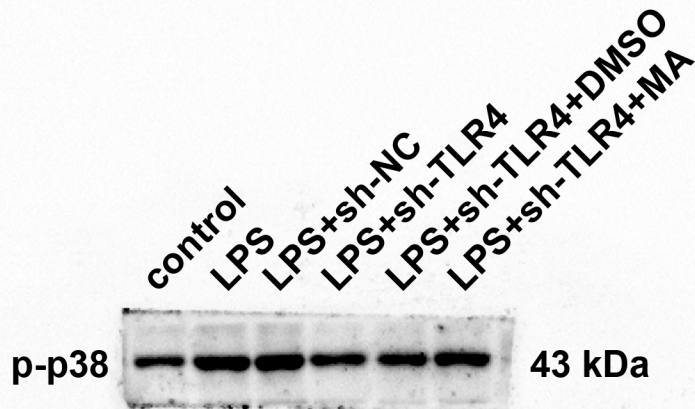

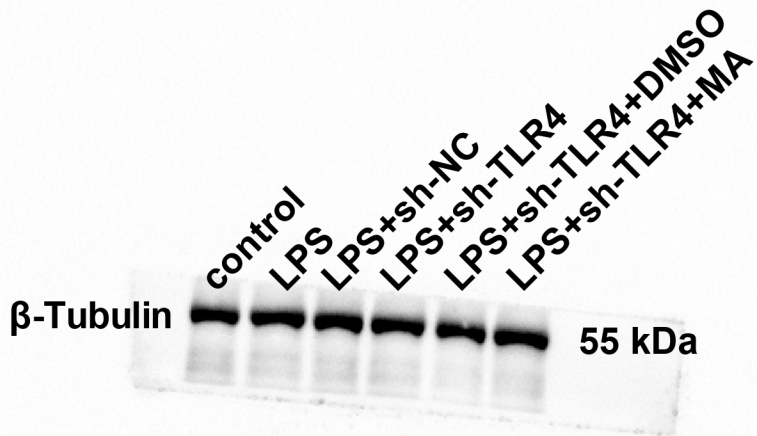

5B

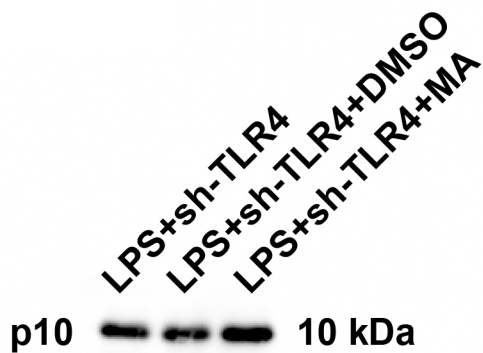

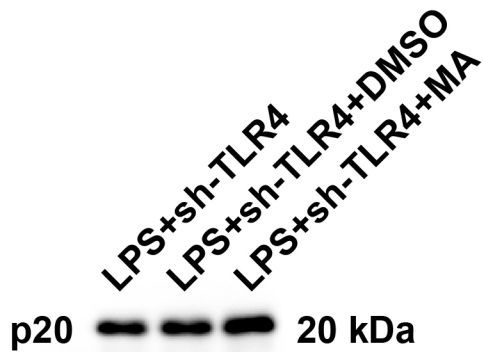

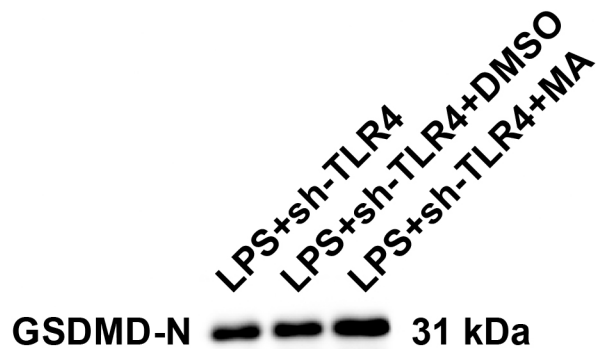

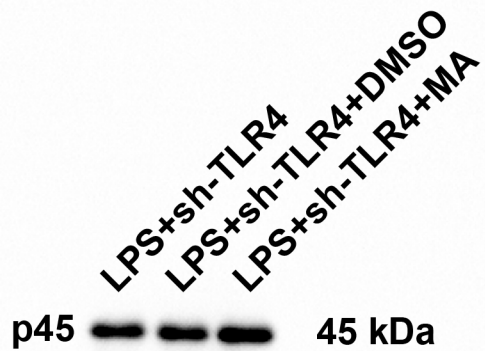

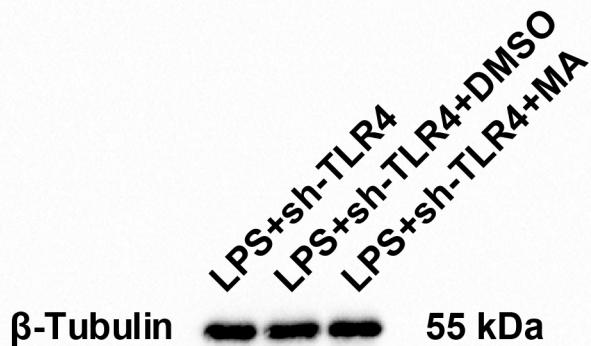

6A

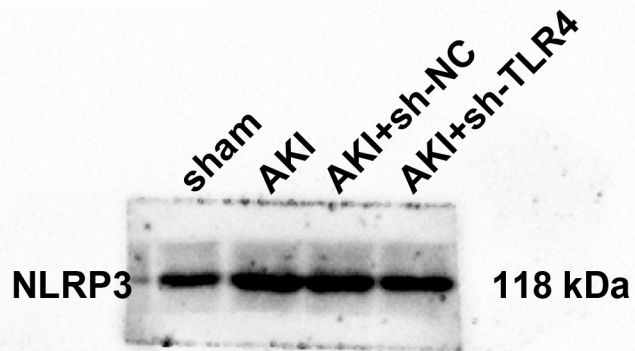

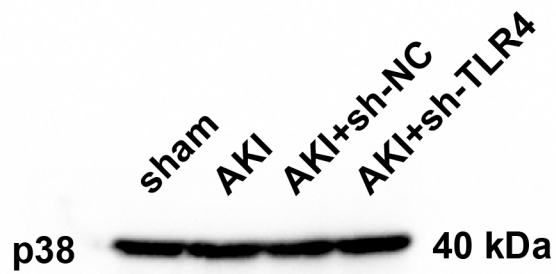

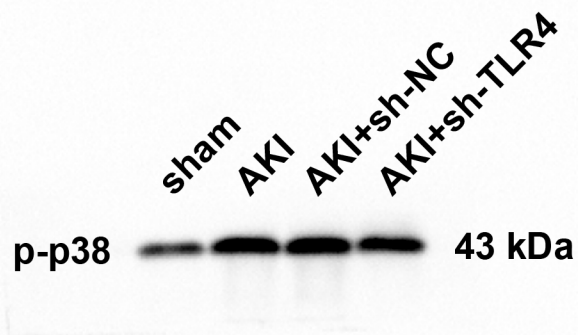

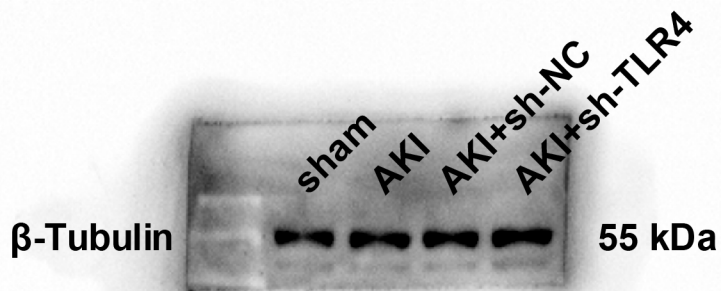

Supplement: Supplementary file 1 — Supplementary Material 1 [file 10863_2023_9972_MOESM1_ESM.pdf]
